# Fermented Dairy Foods: Impact on Intestinal Microbiota and Health-Linked Biomarkers

**DOI:** 10.3389/fmicb.2019.01046

**Published:** 2019-05-24

**Authors:** S. González, T. Fernández-Navarro, S. Arboleya, C. G. de los Reyes-Gavilán, N. Salazar, M. Gueimonde

**Affiliations:** ^1^Area of Physiology, Department of Functional Biology, Faculty of Medicine. University of Oviedo, Oviedo, Spain; ^2^Group Diet, Microbiota and Health, Instituto de Investigación Sanitaria del Principado de Asturias (ISPA), Oviedo, Spain; ^3^Department of Microbiology and Biochemistry of Dairy Products, Instituto de Productos Lácteos de Asturias, Consejo Superior de Investigaciones Científicas (IPLA-CSIC), Villaviciosa, Spain

**Keywords:** fermented foods, yogurt, inflammation, microbiota, oxidative stress

## Abstract

The intake of fermented foods is gaining increasing interest due to their health-promoting benefits. Among them, fermented dairy foods have been associated with obesity prevention, and reduction of the risk of metabolic disorders and immune-related pathologies. Fermented foods could lead to these health benefits by providing the consumer with both easily metabolizable nutrients and beneficial microorganisms. Our aim was to evaluate the relationship between the consumption of fermented dairy products and the intestinal microbiota, serum lipid profile, and the pro-oxidant/inflammatory status. 130 healthy adults were evaluated. Dietary fermented food intake was assessed by an annual food frequency questionnaire (FFQ), including 26 fermented dairy products. Levels of the major phylogenetic types of the intestinal microbiota were determined by qPCR, and concentration of fecal short chain fatty acids were assessed by gas chromatography. Serum glucose and lipid profile, as well as serum malondialdehyde (MDA), C-reactive protein (CRP), and leptin levels were determined by standardized protocols. Among fermented dairy foods, natural yogurt, sweetened yogurt and matured/semi-matured cheese were the most consumed. While natural yogurt consumers showed increased fecal levels of *Akkermansia* with respect to non-consumers, sweetened yogurt intake was associated to lower levels of *Bacteroides*. Serum levels of CRP were also significantly reduced in yogurt consumers. Our results underline the interest in exploring the potential effects of the different yogurt types and the role the microbiota may play in such effects.

## Introduction

Fermented foods have played an important role in human diet since the development of civilization and represent a special feature of some dietary patterns, such as the Mediterranean one. The initial goal of the fermentation process was to prolong the useful-life of some foods and beverages, and improving their safety, digestibility and organoleptic properties, however, nowadays fermented products have become more popular than ever before due to their health-promoting benefits (Şanlier et al., 2017). Fermented dairy foods have received special attention because of their association in epidemiological studies with obesity prevention, and with the reduction on the risk of different diseases, including metabolic disorders, cardiovascular and immune-related diseases or cognitive decline, among others (Guo et al., 2017; Salas-Salvadó et al., 2017; Kok and Hutkins, 2018; Sivamaruthi et al., 2018). Apart from their content of fatty acids, vitamins, and minerals, these products contain bioactive peptides and living microorganisms that could modulate the immune responses and impact on the intestinal microbiota (IM) composition and functionality (Chakrabarti et al., 2014; Severyn and Bhatt, 2018). The human IM is a complex and dynamic community, represented by trillions of microorganisms, that plays an important role in the maintenance of health. Indeed, recent studies have consistently identified disease-specific microbiota signatures in different health disorders (Duvallet et al., 2017). The microbiota of healthy adults is represented mainly by anaerobic bacteria from the Firmicutes and Bacteroidetes phyla (Eckburg et al., 2005). While the genera *Clostridium, Enterococcus, Lactobacillus* and *Faecalibacterium* are predominant within the Firmicutes phylum, others such as *Bacteroides* and *Prevotella* are the most representative of the Bacteroidetes phylum (Eckburg et al., 2005). All of them are present in different proportions depending on the specific microbial composition of each individual. The disruption and alteration of the microbiota may be related to different pathologies and, for this reason, the search for strategies capable of reversing the IM dysbiosis in order to improve the health status of the host has become a key area of interest for the scientific community. In this regard, long-term dietary habits, as well as specific food constituents, such as fiber or phenolics, have been identified as critical drivers of gut microbiota composition (Wu et al., 2011; Fernández-Navarro et al., 2018). Fermented products may also modulate the IM (Kato-Kataoka et al., 2016), however, the association between fermented foods as part of the regular diet and the IM composition has not been sufficiently studied yet (Alvaro et al., 2007; Uyeno et al., 2008). In this regard, a recent work examining the impact of consuming a fermented milk containing microorganisms from the genera *Lactobacillus* and *Bifidobacterium* on the IM has reported a gender-specific increase in the levels of these two bacteria in the feces of volunteers (Lisko et al., 2017). The administration of a probiotic fermented milk, containing *Streptococcus thermophilus*, *Lactobacillus bulgaricus, Lactobacillus acidophilus LA5* and *Bifidobacterium animalis* subsp. *lactis* BB12, during the third trimester of pregnancy has been related with a reduced risk of maternal insulin resistance (Asemi et al., 2013). Yogurt consumption has been associated with immune effects, including a reduced concentration of inflammatory markers in pregnant woman (Asemi et al., 2011). It has also been reported that yogurt modulates both humoral (Meyer et al., 2007) and cellular (Chaves et al., 2011) immunity. Unfortunately, very often observational nutritional studies do not inform us as to whether the positive effect of fermented dairy foods is mediated by the microorganisms present, by some specific components of the product, or by the potential role of some of these products, i.e., yogurt, as a marker of a good overall diet (Kok and Hutkins, 2018). Nevertheless, it is worth underlining that some studies draw attention to the impact yogurt could have, independent of diet (Panahi et al., 2018).

Based on this evidence, it seems reasonable to hypothesize that some of the described beneficial effects of fermented dairy product on several pathologies, such as those affecting the cardiovascular and metabolic systems, might be partly explained by the potential changes induced in the gut microbiota (Marco et al., 2017; Kok and Hutkins, 2018). Thus, in this study we aimed at evaluating the relationship between the consumption of fermented dairy products within the regular diet and the intestinal microbiota. In addition, selected blood markers related with the metabolic profile of the subjects were also analyzed.

## Materials and Methods

This cross-sectional study sample comprised of 130 subjects from the Principality of Asturias Region (Northern Spain). Inclusion criteria were: not being diagnosed with diseases related to intestinal function, not being currently treated with corticoids, nor having consumed pro- and prebiotic supplements or antibiotics during the previous month. Participants were mentally and physically able to participate in the study and gave written informed consent. Ethical approval was obtained from the Bioethics Committee of CSIC and from the Regional Ethics Committee for Clinical Research of the Principality of Asturias in compliance with the Declaration of Helsinki of 1964. All experiments were carried out in accordance with approved guidelines and regulations.

### Blood Biochemical Analysis

Blood samples were kept on ice and centrifuged (1000 ×*g*, 15 min) within 2–4 h after collection. Plasma and serum aliquots were kept at -20°C until analyses were performed. Plasma glucose, cholesterol, and triglycerides were determined by standard methods. Serum levels of C-reactive protein (CRP) were assessed using a CRP Human Instant ELISA kit (eBioscience, San Diego, CA, United States), and those of malondialdehyde (MDA) with a colorimetric assay of lipid peroxidation (Bioxytech LPO-586, Oxis International SA, Paris, France); the within-run coefficient of variation ranged from 1.2 to 3.4%, depending on the concentration of MDA (Gerard-Monnier et al., 1998). Serum leptin was measured by a sensitive ELISA test (Human Leptin ELISA Development Kit, PeproTech Inc., Rocky Hill, CT, United States); the detectable concentration range was 63–4000 pg/mL and the intra-assay and inter-assay coefficients of variation were 5.21 and 5.20%, respectively.

### Microbial Analysis

Fecal samples were immediately frozen at -20°C and transported to the laboratory. For analyses fecal samples were melted, weighed, diluted 1/10 in sterile PBS, and homogenized at full-speed in a LabBlender 400 Stomacher (Seward Medical, London, United Kingdom) for 4 min. The samples were then centrifuged and the supernatant was taken for SCFA analyses whereas the fecal pellet was used for DNA extraction using the QIAamp DNA stool mini kit (Qiagen, Hilden, Germany) as previously described (Arboleya et al., 2012).

Quantification of different bacterial populations was performed with a 7500 Fast Real-Time PCR System (Applied Biosystems, Foster City, CA, United States) using SYBR Green PCR Master Mix (Applied Biosystems), and covered the major bacterial groups present in the gut microbial ecosystem. One microliter of template fecal DNA (∼5 ng) and 0.2 μM of each primer were added to the 25 μL reaction mixture. PCR cycling consisted of an initial cycle of 95°C 10 min, followed by 40 cycles of 95°C 15 s, and 1 min at the appropriate primer-pair temperature. The number of cells was determined by comparing the Ct values obtained from a standard curve. Fecal DNA extracts were analyzed and the mean quantity per gram of fecal wet weight was calculated as indicated elsewhere (Arboleya et al., 2012).

The analysis of SCFA was performed by gas chromatography in system composed of a 6890N GC injection module (Agilent Technologies Inc., Palo Alto, CA, United States) connected to a flame injection detector (FID) and a mass spectrometry (MS) 5973N detector (Agilent), as described previously (Arboleya et al., 2016).

### Nutritional Assessment

Dietary intake was assessed in a personal interview by means of an annual semi-quantitative food frequency questionnaire (FFQ) method validated in previous studies (Cuervo et al., 2014). The FFQ was organized by food groups and open-ended, allowing foods consumed by the subject and not present in the questionnaire to be recorded. Among the dairy products group, 26 items were listed, including the three major fermented food groups: yogurt, cheese, and fermented milk. Food intake was analyzed for energy, macronutrients, and total dietary fiber content by using the nutrient Food Composition Tables developed by CESNID (Centro de Enseñanza Superior de Nutrición Humana y Dietética [CESNID], 2008). Additionally, the following fiber components were ascertained using (Marlett and Cheung, 1997) food composition tables: soluble fiber, insoluble fiber based on the enzymatic-chemical method developed by Theander and Westerlund (1986).

Height and weight were recorded after an overnight fast, using the standardized procedures described previously (Fernández-Navarro et al., 2017) for BMI [weight (Kg)/height (m^2^)]. Body fat percentage was measured by bioelectrical impedance (BIA) with ± 1% variation (Tanita Corporation of America, Inc., Arlington Heights, IL, United States).

### Statistical Analysis

Statistical analysis was performed using the IBM SPSS program version 22.0 (IBM SPSS, Inc., Chicago, IL, United States). Goodness of fit to the normal distribution was analyzed by means of the Kolmogorov-Smirnov test. Categorical variables were summarized with percentages while continuous variables were summarized using mean and standard deviations. The chi-squared test and independent samples *t*-test were used for group comparisons where appropriate. Pearson bivariate correlation was used to investigate linear association between the intake of total fermented dairy products and each subgroup, with the major food groups consumed in the diet and to describe the relationship between the consumption of fermented dairy foods with fecal microbial levels and serum health-related biomarkers. Heatmap was generated under R version 3.5.1 package heatmap.2. The conventional probability value for significance (0.05) was used in the interpretation of results.

## Results

The general characteristics of the study sample defined a group of 130 healthy adults with a mean age of 58.2 ± 17.1 years, and a moderate overweight (Table 1). 12.3% of the sample were smokers, and 55% lived sedentary lifestyles. To avoid potential confounding factors, like age or gender, these variables were included as covariables in any further analysis.

**Table 1:** General description of the study sample.

			Total	Gender	
			*n* = 130	Male *n* = 38	Female *n* = 92
Age (y)			58.18 ± 17.10	57.95 ± 17.20	58.28 ± 17.20
BMI (kg/m^2^)			27.04 ± 4.40	27.73 ± 3.19	26.75 ± 4.80
Sedentary (%)			55.3	42.1	**61.0^∗^**
Current smoker (%)			12.3	15.8	10.9
Energy intake (Kcal)			1919.34 ± 552.4	2079.39 ± 652.48	1853.23 ± 494.4*
Total lipids (g/day)^a^			80.04 ± 28.14	76.35 ± 30.47	81.56 ± 27.18
	PUFA		14.03 ± 7.67	13.99 ± 8.20	14.05 ± 7.67
	MUFA		32.73 ± 15.66	31.00 ± 19.18	33.45 ± 14.03
	SFA		26.84 ± 10.12	25.16 ± 7.27	27.54 ± 11.11
Total protein (g/day)^a^			80.01 ± 26.71	84.43 ± 32.10	90.90 ± 24.31*
	Animal protein		59.52 ± 21.91	53. 36 ± 25.72	62.06 ± 20.22*
	Vegetal protein		27.18 ± 10.01	29.30 ± 12.38	26.31 ± 8.35*
Total carbohydrates (g/day)^a^			200.22 ± 66.37	210.41 ± 76.70	196.01 ± 58.79*
Total fiber (g/day)^a^			19.94 ± 7.56	19.89 ± 7.75	19.96 ± 7.45
	Soluble fiber		2.57 ± 1.15	2.53 ± 1.27	2.58 ± 1.10
	Insoluble fiber		12.85 ± 5.56	12.33 ± 5.81	13.06 ± 12.65
Total dairy products (g/day)^a^			388.23 ± 219.24	331.29 ± 208.87	411.75 ± 222.15
	Milk and non-fermentable dairies (g/day)		255.37 ± 183.54	223.78 ± 176.60	268.42 ± 186. 20
	Fermented dairy products (g/day)		129.46 ± 111.29	101.59 ± 110.70	140.98 ± 111.0
	Yogurt (g/day)		96.46 ± 102.19	85.81 ± 103.35	100.86 ± 102.06
		Natural yogurt	77.82 ± 102.38	69.74 ± 102.44	81.16 ± 102.78
		Sweetened yogurt	18.64 ± 51.40	16.08 ± 43.02	19.70 ± 54.69
	Cheese (g/day)		24.92 ± 35.56	13.92 ± 21.09	29.47 ± 39.79*
		Matured/semi-matured cheese	13.83 ± 22.29	11.83 ± 19.14	14.65 ± 26.57
		Fresh cheese	11.16 ± 26.48	2.04 ± 12.23	14.93 ± 30.03*
	Fermented milk (ml/day)		8.08 ± 33.70	1.86 ± 16.22	10.65 ± 38.54


Results are presented as estimated marginal mean ± SD and percentage (%). ^a^Univariate analysis adjusted by total energy intake. PUFA, polyunsaturated fatty acids; MUFA, monounsaturated fatty acids, SFA, saturated fatty acids. Variables included in natural yogurt: whole natural yogurt, skimmed natural yogurt and lactose-free natural yogurt. Variables included in sweetened yogurt: whole flavored yogurt, whole sweetened yogurt, whole yogurt with fruits, skimmed flavored yogurt, skimmed sweetened yogurt, skimmed yogurt with fruits and Greek yogurt. Variables included in matured/semi-matured cheese: blue cheese, matured/semi-matured cow cheese, matured/semi-matured goat cheese and processed cheese. Variables included in fresh cheese: fresh goat cheese and fresh cow cheese. Variables included in fermented milks: natural milk with Bifidobacterium, milk with Bifidobacterium and fruit, natural milk with Lactobacillus and milk with Bifidobacterium and sterols*p value ≤ 0.05. Bold characters indicate statistically significant differences (p < 0.05).

**FIGURE 1:**
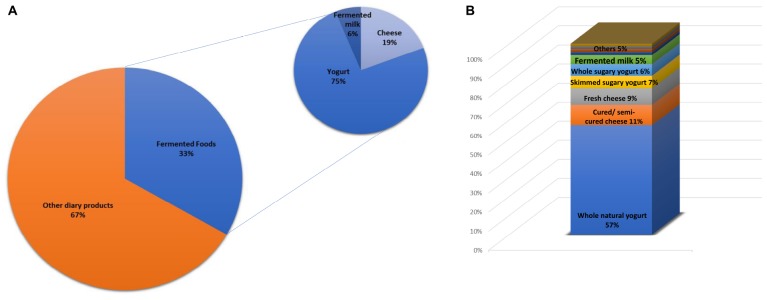
**(A)** Contribution (%) of the fermented dairy foods and main subgroups to the total intake of dairy products. **(B)** Intake proportion of detailed fermented dairy foods in the sample.

**FIGURE 2:**
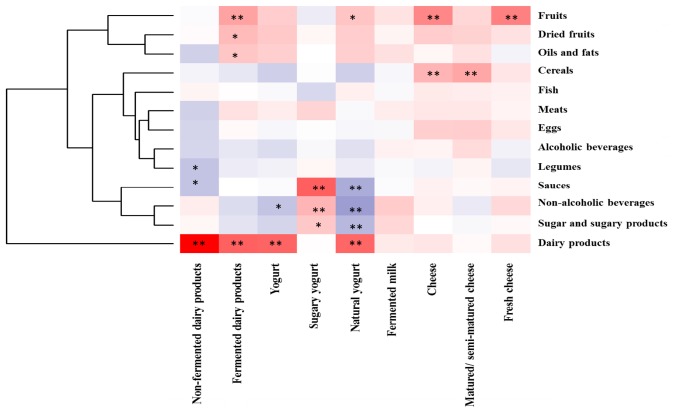
Pearson correlation between the intake of major food groups (g/day) with fermented dairy foods (g/day) in the sample. Columns correspond to main fermented dairy products whereas rows correspond to food groups. Blue and red colors denote negative and positive association, respectively. The intensity of the color represents the degree of association between the fermented dairies consumed in the sample and major food groups in the diet. Asterisks indicate significant associations: ^∗^*p* < 0.05; ^∗∗^*p* ≤ 0.01.

The total consumption of milk and dairy products (388.23 g/day) (Table 1) corresponded, in 33% of the sample, to the intake of fermented dairy foods, mainly yogurt and cheese (75 and 19%, respectively), as shown in Figure 1A. Among fermented dairy foods, natural yogurt (77.82 ± 102.38 g/day), sweetened yogurt (18.64 ± 51.40 g/day) and matured/semi-matured cheese (13.83 ± 22.29 g/day) were the most consumed (Table 1). Among them, natural yogurt was the main contributor (Figure 1B). The relationship between fermented dairy products and major food groups from the diet is shown in Figure 2. The consumption of fermented dairy foods presented a significant positive association with the intake of total dairy products, oils and fats, and dried fruits. In more detail, yogurt was negatively related to the intake of non-alcoholic beverages, and the consumption of cheese presented a direct relation with cereals, and fruits from the regular diet. Focusing on yogurt types, natural yogurt was directly related to the intake of dairy products and fruits, and negatively associated with sugars, sauces and non-alcoholic beverages; on the contrary, the intake of sweetened yogurt was positively related to these latter food groups (Figure 2). In the case of cheese, matured/semi-matured cheese consumption presented a positive relationship with the intake of cereals, while fresh cheese did it with fruits. Fermented milk has not been significantly associated with the intake of none of the other assessed food groups (Figure 2).

Regarding fecal microbial composition, natural yogurt consumers showed significantly higher fecal levels of *Akkermansia,* and sweetened yogurt consumers displayed significantly lower fecal levels of *Bacteroides* than non-consumers. Moreover, cheese consumers (considering all types jointly) presented significantly higher levels of the major fecal SCFA, acetate, propionate and butyrate, whereas the consumers of fresh cheese specifically presented higher levels of propionate and butyrate than non-consumers (Table 2).

**Table 2:** Differences in the concentration of major microbial groups and short chain fatty acids according to the intake of the different types of fermented dairy foods consumed by the sample.

	Yogurt (g/day)		Natural Yogurt (g/day)		Sweetened Yogurt (g/day)		Cheese (g/day)		Matured/semi-matured cheese (g/day)		Fresh cheese (g/day)		Fermented milk (mg/day)	
	Non-consumers	Consumers	Non-consumers	Consumers	Non-consumers	Consumers	Non-consumers	Consumers	Non-consumers	Consumers	Non-consumers	Consumers	Non-consumers	Consumers
	(*n* = 27)	(*n* = 103)	(*n* = 50)	(*n* = 80)	(*n* = 106)	(*n* = 24)	(*n* = 27)	(*n* = 103)	(*n* = 47)	(*n* = 83)	(*n* = 86)	(*n* = 44)	(*n* = 122)	(*n* = 8)
**Microbial target (log no. cells per gram of feces)**			
*Akkermansia*	4.9 ± 2.4	5.6 ± 2.3	4.9 ± 2.3	5.8 ± 2.2*	5.6 ± 2.3	5.0 ± 2.2	5.4 ± 2.5	5.5 ± 2.3	5.2 ± 2.3	5.6 ± 2.4	5.6 ± 2.4	5.2 ± 2.3	5.6 ± 2.4	4.6 ± 2.5
*Bacteroides* group	8.3 ± 2.0	8.3 ± 1.8	8.0 ± 2.0	8.5 ± 1.7	8.5 ± 1.7	7.6 ± 1.9*	8.9 ± 1.0	8.3 ± 1.8	8.2 ± 2.0	8.4 ± 1.7	8.4 ± 1.6	8.3 ± 2.2	8.4 ± 1.9	8.1 ± 1.1
*Bifidobacterium sp*	7.4 ± 1.6	7.4 ± 1.7	7.3 ± 1.7	7.7 ± 1.7	7.6 ± 1.7	7.3 ± 1.8	7.6 ± 1.7	7.5 ± 1.7	7.6 ± 1.8	7.5 ± 1.7	7.5 ± 1.7	7.6 ± 1.8	7.6 ± 1.8	7.5 ± 0.5
*Clostridium* cluster XIVa	7.0 ± 2.4	7.0 ± 2.3	6.9 ± 2.7	7.0 ± 2.1	7.0 ± 2.2	6.9 ± 3.0	6.9 ± 2.3	7.0 ± 2.3	7.1 ± 2.2	6.9 ± 2.4	6.9 ± 2.3	7.2 ± 2.3	7.1 ± 2.3	6.0 ± 2.8
*Lactobacillus* group	5.5 ± 1.6	5.8 ± 1.9	5.7 ± 1.8	5.8 ± 1.8	5.7 ± 1.8	5.9 ± 2.0	6.2 ± 2.2	5.6 ± 1.7	6.0 ± 2.1	5.6 ± 1.7	5.9 ± 1.8	5.6 ± 1.9	5.8 ± 1.9	5.6 ± 1.1
*Faecalibacterium prausnitzii*	6.8 ± 1.5	6.8 ± 1.8	6.8 ± 1.6	6.8 ± 1.7	6.8 ± 1.7	6.9 ± 1.7	6.3 ± 2.4	7.0 ± 1.5	6.5 ± 2.1	7.0 ± 1.4	6.8 ± 1.8	6.8 ± 1.6	6.9 ± 1.8	6.7 ± 0.9
**SCFA concentration (mM)**			
Acetate	33.2 ± 15.9	36.5 ± 19.6	37.5 ± 18.2	34.7 ± 17.9	34.2 ± 17.5	42.6 ± 17.1*	28.9 ± 14.5	37.6 ± 18.5*	34.6 ± 20.7	36.5 ± 17.4	34.4 ± 17.4	38.4 ± 20.4	36.4 ± 18.8	27.4 ± 19.9
Propionate	12.5 ± 6.5	13.1 ± 7.6	13.1 ± 7.2	13.0 ± 7.3	12.8 ± 7.1	13.8 ± 7.6	9.8 ± 5.9	13.9 ± 7.2*	12.6 ± 7.7	13.3 ± 7.1	12.1 ± 7.3	14.7 ± 7.1*	13.2 ± 7.4	10.6 ± 7.6
Butyrate	11.7 ± 9.0	10.0 ± 7.1	11.2 ± 7.2	9.8 ± 7.6	10.4 ± 8.1	10.1 ± 4.4	7.7 ± 5.7	11.1 ± 7.7*	9.8 ± 7.0	10.7 ± 7.8	9.4 ± 6.9	12.1 ± 8.4*	10.5 ± 7.7	8.7 ± 5.6


Results from univariate analyses, adjusted by age and gender, were presented as mean ± standard deviation. Bacteroides group, Bacteroides-Prevotella-Porphyromonas; SCFA, Short chain fatty acids. Variables included in natural yogurt: whole natural yogurt, skimmed natural yogurt and lactose-free natural yogurt. Variables included in sweetened yogurt: whole flavored yogurt, whole sweetened yogurt, whole yogurt with fruits, skimmed flavored yogurt, skimmed sweetened yogurt, skimmed yogurt with fruits and Greek yogurt. Variables included in matured/semi-matured cheese: blue cheese, matured/semi-matured cow cheese, matured/semi-matured goat cheese and processed cheese. Variables included in fresh cheese: fresh goat cheese and fresh cow cheese. Variables included in fermented milks: natural milk with Bifidobacterium, milk with Bifidobacterium and fruit, natural milk with Lactobacillus and milk with Bifidobacterium and sterols. *p value ≤ 0.05. Bold characters indicate statistically significant differences (p < 0.05).

Delving into the impact of fermented dairy foods on health status, the association between them and serum health biomarkers was analyzed. While the intake of yogurt, especially natural yogurt, showed a direct association with LDL/HDL ratio values, serum CRP was significantly lower in yogurt consumers (5.5 ± 10.5 vs. 2.1 ± 4.6 mg/L). Moreover, natural yogurt was associated with the oxidant status, the consumers of this product showing also lower levels of serum MDA (2.80 ± 1.33 vs. 2.28 ± 0.59 μM) than non-consumers (Table 3). The intake of cheese and its different types or fermented milk did not show any association with any health-related biomarker (Table 3).

**Table 3:** Differences in anthropometric parameters and mean concentrations of serum health related biomarkers according to the intake of the different types of fermented dairy foods consumed by the sample.

	Yogurt (g/day)		Natural Yogurt (g/day)		Sweetened Yogurt (g/day)		Cheese (g/day)		Matured/semi-matured cheese (g/day)		Fresh cheese (g/day)		Fermented milk (mg/day)	
	Non-consumers	Consumers	Non-consumers	Consumers	Non-consumers	Consumers	Non-consumers	Consumers	Non-consumers	Consumers	Non-consumers	Consumers	Non-consumers	Consumers
	(*n* = 27)	(*n* = 103)	(*n* = 50)	(*n* = 80)	(*n* = 106)	(*n* = 24)	(*n* = 27)	(*n* = 103)	(*n* = 47)	(*n* = 83)	(*n* = 86)	(*n* = 44)	(*n* = 122)	(*n* = 8)
BMI (kg/m^2^)	27.0 ± 4.5	27.0 ± 4.4	27.5 ± 4.9	26.7 ± 4.1	26.8 ± 4.2	27.9 ± 5.2	26.7 ± 5.0	27.1 ± 27.1	27.2 ± 4.7	26.93 ± 4.2	27.0 ± 4.1	27.1 ± 4.9	27.1 ± 4.4	25.9 ± 3.7
Body fat (%) n63	37.3 ± 10.6	35.1 ± 12.0	38.1 ± 12.4	33.9 ± 11.3	34.8 ± 11.1	38.7 ± 13.9	34.7 ± 12.8	35.6 ± 11.6	35.7 ± 13.4	35.44 ± 11.0	35.1 ± 11.1	36.1 ± 12.1	-	-
**Blood parameters**			
Glucose (mg/dL)	96.0 ± 9.6	100.3 ± 20.3	96.7 ± 9.9	101.0 ± 22.1	99.5 ± 19.9	98.7 ± 10.5	97.2 ± 19.7	100.0 ± 18.6	97.0 ± 16.2	101.0 ± 20.2	100.5 ± 21.4	97.1 ± 11.1	99.6 ± 19.1	96.2 ± 11.5
Triglycerides (mg/dL)	115.4 ± 70.1	116.6 ± 60.0	117.0 ± 79.0	116.1 ± 50.2	115.9 ± 55.3	119.2 ± 90.9	126.9 ± 60.7	113.5 ± 62.2	117.7 ± 65.1	115.6 ± 60.3	118.4 ± 63.8	112.34 ± 57.7	117.5 ± 62.4	94.7 ± 47.8
Total cholesterol (mg/dL)	212.0 ± 40.8	212.4 ± 40.3	207.6 ± 37.6	2015.1 ± 41.6	214.2 ± 41.3	202.6 ± 33.6	201.4 ± 47.6	215.3 ± 38.0	206.1 ± 42.8	216.2 ± 38.5	209.7 ± 41.6	217.7 ± 37.3	211.6 ± 40.1	226.4 ± 43.8
LDL/HDL ratio	2.1 ± 0.8	2.6 ± 0.9*	2.2 ± 0.8	2.7 ± 0.9*	2.5 ± 0.9	2.4 ± 0.9	2.4 ± 0.9	2.5 ± 0.9	2.4 ± 0.9	2.6 ± 0.9	2.6 ± 0.9	2.4 ± 0.8	2.5 ± 0.9	2.0 ± 1.0
Leptin (ng/mL)	11.1 ± 7.9	9.9 ± 6.5	11.2 ± 6.9	9.6 ± 6.5	9.9 ± 6.8	10.9 ± 6.0	9.4 ± 7.4	10.3 ± 6.5	9.7 ± 7.1	10.2 ± 6.4	9.9 ± 6.8	10.6 ± 6.3	–	–
CRP (mg/L)	5.5 ± 10.5	2.1 ± 4.6*	4.2 ± 9.1	2.0 ± 3.9	2.8 ± 5.9	1.6 ± 7.3	3.4 ± 4.2	2.4 ± 6.5	3.9 ± 7.0	1.9 ± 5.4	2.5 ± 5.6	3.1 ± 7.1	–	–
MDA (μM)	2.6 ± 1.7	2.4 ± 0.6	2.8 ± 1.3	2.3 ± 0.6*	2.4 ± 0.87	2.9 ± 0.8	2.7 ± 0.5	2.3 ± 1.0	2.6 ± 1.1	2.3 ± 0.69	2.5 ± 0.6	2.3 ± 1.3	–	–


Results from univariate analyses, adjusted by age and gender, were presented as mean ± standard deviation. CRP, C-reactive protein; MDA, malondialdehyde. Variables included in natural yogurt: whole natural yogurt, skimmed natural yogurt and lactose-free natural yogurt. Variables included in sweetened yogurt: whole flavored yogurt, whole sweetened yogurt, whole yogurt with fruits, skimmed flavored yogurt, skimmed sweetened yogurt, skimmed yogurt with fruits and Greek yogurt. Variables included in matured/semi-matured cheese: blue cheese, matured/semi-matured cow cheese, matured/semi-matured goat cheese and processed cheese. Variables included in fresh cheese: fresh goat cheese and fresh cow cheese. Variables included in fermented milks: natural milk with Bifidobacterium, milk with Bifidobacterium and fruit, natural milk with Lactobacillus and milk with Bifidobacterium and sterols. (–) Data not available. *p value ≤ 0.05. Bold characters indicate statistically significant differences (p < 0.05).

## Discussion

The present study is a pioneer report analyzing the relationship between the intake of fermented dairy foods within the regular diet, the gut microbial profile and health related biomarkers, considering the subject’s global diet. Previous studies identified diets rich in fruits, vegetables or whole grains as critical modulators of the gut microorganisms, based on their content in fibers, phenolic compounds and prebiotics (Cuervo et al., 2014; Fernández-Navarro et al., 2018). However, the association between the different live microorganisms provided by the diet within the intestinal ecosystem offers a novel way to look into gut microbiota composition and its metabolic activity (Kok and Hutkins, 2018). In this regard, our results showed that, among the fermented dairy products assessed, yogurt was the product which showed higher ability to modulate the fecal microbiota. Interestingly, while the consumption of natural yogurt was directly associated with *Akkermansia* levels, the sweetened yogurt was inversely related with *Bacteroides* counts. The consumption of yogurt has been correlated with a good quality diet and some studies pointed out differences among yogurt types (Gómez-Gallego et al., 2018). A Danish cohort study suggested that consumption of whole-fat yogurt instead of low-fat products may be associated with a lower risk of type-2 diabetes (Ibsen et al., 2017). In the present sample, unfortunately, the low consumption of skimmed yogurt (consumed by only 6 out of the 80 volunteers consuming natural yogurt) precluded a skimmed vs. whole-fat comparison, however, it is worth mentioning that we have observed differences among the yogurt types assessed (natural vs. sweetened) with regards to the microbiota profile. These results underline the need for a full subcategorization of yogurt types in intervention and epidemiological studies, since different types may differ in their effects on health.

Given the descriptive nature of our study, we are not able to elucidate the mechanism of action explaining the observed associations. In spite of the lack of information about the modulation of intestinal *Akkermansia* in humans, recent research in mice treated with antibiotics has reported an increase in this bacterial group after the administration of a probiotic mix of *Lactobacillus* (Shi et al., 2018). Therefore, it may be plausible that the intake of such microorganisms, present in yogurt, might play a role in this association (Hill et al., 2014; Rezac et al., 2018). At this point, it should be mentioned that since labels of products do not provide information about the viable microorganisms present, we cannot know the exact amount and specific strains consumed by the study sample. According to the CODEX regulation (CODEX STAN 243-2003), yogurt must include a minimum bacterial counts of 10^7^ cfu per gram from the symbiotic cultures of *Streptococcus thermophilus* and *Lactobacillus bulgaricus.* This, according to the intake data obtained, would correspond with intakes between 5 × 10^8^ and 10^9^ bacterial cells/day of each of these microorganisms. Nevertheless, although as shown in this study these levels can be easily reached within the context of a normal diet, it is also true that in interventional studies higher levels have been often used (Meyer et al., 2007; Asemi et al., 2011).

Results from intervention studies, both in animals and humans, have shown that the increase in *Akkermansia muciniphila* is associated with lower adiposity and a better metabolic status, suggesting this microorganism could be a potential candidate for obesity control (Everard et al., 2013; Dao et al., 2016; Rodríguez-Carrio et al., 2017). In the current study, we found that natural yogurt consumers presented not only higher intestinal *Akkermansia* levels with respect to non-consumers, but also a “healthier metabolic profile” based on lower inflammation and serum lipid peroxidation, measured through serum CRP and MDA. These immune variables have been reported to be moderately reduced in intervention studies with probiotic yogurt by other authors (Mohamadshahi et al., 2014; Burton et al., 2017). These findings are coherent with recent data from the Kuopio Ischaemic Heart Disease Risk Factor Study showing a cardiovascular protective effect in men consuming fermented dairy products (Koskinen et al., 2018), and with several epidemiological studies supporting a protective role of fermented dairy products against the chronic “low-grade” inflammation associated with the metabolic syndrome and related diseases (Baothman et al., 2016; Kim et al., 2017; Salas-Salvadó et al., 2017). Despite the values of LDL/HDL ratio in our sample were higher for yogurt consumers than for non-consumers, these are far from the established levels of atherogenic risk (>4.5). It is also important to underline that, in contrast to some of the previous studies (Asemi et al., 2013), age and gender have been introduced as covariates in the analyses performed in our study, and global diet has been determined.

Fermented dairy foods may present nutritional properties independent of the presence of microorganisms, as seems to occur with the sweetened yogurts. Although the lower levels of *Bacteroides* observed in the consumers of sweetened yogurt in our sample could be *a priori* surprisingly; this result is in consonance with previous reports indicating a reduction in the intestinal level of *Bacteroides* associated with the consumption of certain sweeteners such as sucralose (Uebanso et al., 2017). Therefore, it could be interesting to examine if the addition of additives (flavors, sweeteners, etc.) to traditionally considered healthy products, such as yogurt, could influence on the gut microbiota and, therefore, on the health status of the host.

No statistical differences were found in the levels of intestinal microbial groups as related to cheese consumption. However, cheese consumers showed higher fecal concentrations of the major SCFA. These compounds have been widely related with different metabolic effects, directly modulating host health through a range of tissue-specific mechanisms (den Besten et al., 2013; Rios-Covian et al., 2016; Uebanso et al., 2017). From a nutritional point of view, differences in the relationship with health may be expected depending on the types of cheese considered. Notwithstanding, we have not observed differences in our sample in health-related parameters according to cheese intake.

It is also important to be aware that this study contains some limitations. As mentioned before, although the FFQ has been carried out with a high grade of detail, it has not been possible to collect information on the specific microbial strains contained in the products. On the other hand, even though the multivariate models were adjusted by age and gender, we cannot rule out possible residual confounders often present in this sort of study. In spite of this, the present work has the strength of being conducted within the context of the habitual and global dietary pattern of the volunteers, and points out natural yogurt as a healthy product that, as previously suggested (Gómez-Gallego et al., 2018), should have a more visible role in dietary recommendations and guidelines. Our data suggests that fermented dairy products in general, and yogurt in particular, could be a key element affecting the relationship between diet and health by means of the modulation of gut microbial composition and functionality.

## Data Availability

The datasets generated for this study are available on request to the corresponding author.

## Ethics Statement

This study was carried out in accordance with the recommendations of the Bioethics Committee from CSIC and the Regional Bioethics Committee from the Principality of Asturias (Spain) with written informed consent from all subjects. All subjects gave written informed consent in accordance with the Declaration of Helsinki. The protocol was approved by the Bioethics Committee from CSIC and the Regional Bioethics Committee from the Principality of Asturias (Spain).

## Author Contributions

MG and SG had the primary responsibility in the study design and protocol development, and confirm that they had full access to the data in the study and final responsibility for the decision to submit for publication and drafted the manuscript. SG and TF-N were involved in data collection and contributed to the dietary and nutritional data analysis and interpretation. CR-G, SA, and NS conducted the microbial analysis and data processing and supervised the execution of the study and data analysis. All authors critically reviewed the manuscript and approved the final version submitted for publication.

## Conflict of Interest Statement

The authors declare that the research was conducted in the absence of any commercial or financial relationships that could be construed as a potential conflict of interest.
